# Epidural Anesthesia Complicated by Subdural Hygromas and a Subdural Hematoma

**DOI:** 10.1155/2016/5789504

**Published:** 2016-08-29

**Authors:** Christine Vien, Paul Marovic, Brendan Ingram

**Affiliations:** ^1^Monash Health, 246 Clayton Road, Clayton, Melbourne, VIC 3168, Australia; ^2^Alfred Health, 55 Commercial Road, Melbourne, VIC 3004, Australia

## Abstract

Inadvertent dural puncture during epidural anesthesia leads to intracranial hypotension, which if left unnoticed can cause life-threatening subdural hematomas or cerebellar tonsillar herniation. The highly variable presentation of intracranial hypotension hinders timely diagnosis and treatment. We present the case of a young laboring adult female, who developed subdural hygromas and a subdural hematoma following unintentional dural puncture during initiation of epidural anesthesia.

## 1. Introduction

Inadvertent dural puncture during epidural anaesthesia leads to intracranial hypotension, which if left unnoticed can cause life-threatening complications such as subdural hematomas and cerebellar tonsillar herniation [1, 2]. The highly variable presentation of intracranial hypotension hinders timely diagnosis and treatment.

## 2. Case Presentation

A twenty-seven-year-old otherwise healthy nulliparous patient requested epidural anesthesia for pain relief during spontaneous labor.

Following informed consent and using an aseptic technique, an 18 g Tuohy needle was inserted into the L3-4 epidural space, guided by a loss of resistance to normal saline. Unfortunately, the thecal sac was breached and the needle was immediately withdrawn. A second attempt, through the L2-L3 interspinous space, resulted in the successful placement of an epidural catheter and this was confirmed with a test dose of 10 mL of 0.2% ropivacaine. Further analgesia was provided via patient controlled epidural analgesia (PCEA) using 5 mL of 0.125% bupivacaine with a lockout of 15 minutes, as per the institution's protocol. There was no evidence of a high block. Six hours after the initiation of epidural analgesia, the patient required instrumental delivery with Kielland's rotational forceps.

The patient developed a mild, intermittent, nonpostural headache on day one following delivery but was able to continue caring for her newborn child. Her neurological examination and vital signs were normal. The symptoms were not indicative of a Postdural Puncture Headache (PDPH) and she was treated with intravenous hydration and oral analgesia.

On day two, the patient's headache became persistent and postural, and she developed nausea and vomiting. This was attributed to PDPH and she was informed of the potential treatments including autologous blood patching. She declined the blood patch and wished to continue with conservative management of paracetamol, ibuprofen, metoclopramide, and ondansetron with reasonable control. On day three, the Medical Emergency Team urgently attended the patient's bedside due to the onset of bradycardia (heart rate of forty beats per minute) in the setting of severe headache and vomiting. The patient was promptly investigated with Computed Tomography (CT).

**Figure 1:**
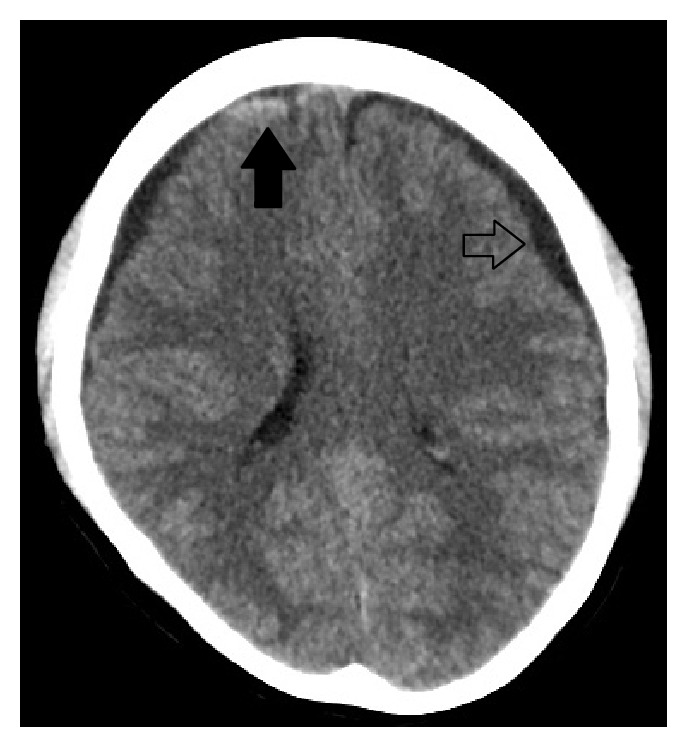
Nonintravenous contrast enhanced brain CT demonstrates bilateral CSF-density subdural hygromas (left subdural hygroma labelled with an open arrow) and a hyperdense acute right frontal subdural hematoma (solid arrow).

**Figure 2:**
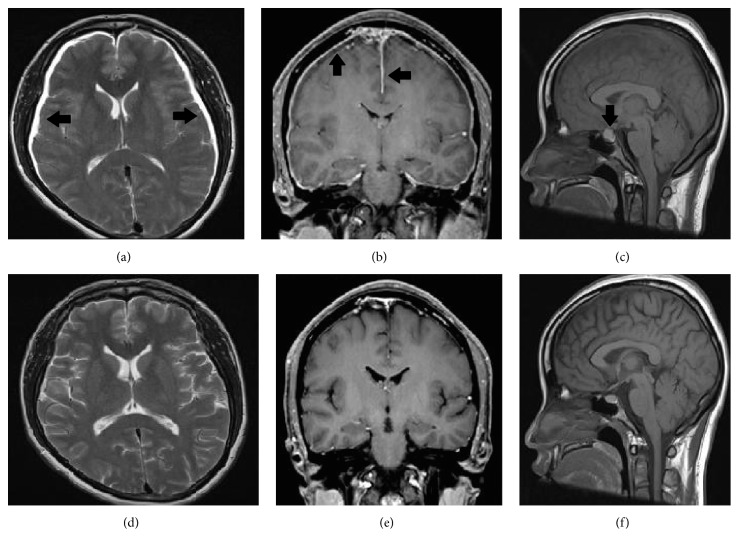
(a) Axial T2 weighted sequence demonstrates bilateral CSF-intensity subdural hygromas (arrows). (b) Coronal T1 weighted gadolinium enhanced sequence demonstrates pachymeningeal thickening and enhancement (arrows). (c) Sagittal T1 weighted sequence demonstrates pituitary gland enlargement. (d)–(f) Posttreatment MRI examination demonstrates complete radiological resolution.

Brain CT demonstrated bilateral cerebral convexity subdural hygromas and a small right frontal subdural hematoma (Figure 1), while a head CT venogram was unremarkable. The patient also underwent a brain MRI, which demonstrated further classical signs of intracranial hypotension, namely, slit-like lateral ventricles, an enlarged pituitary gland, and aseptic pachymeningitis (Figure 2)  [3].

On day four, an epidural blood patch was performed without complication using 25 mL of autologous blood, resulting in rapid relief of the patient's headache.

A follow-up brain MRI was performed one month later, which demonstrated complete resolution of the subdural hygromas (Figure 2). The patient was symptom-free.

## 3. Discussion

Postpartum headache is extremely common, reportedly occurring in up to 80% of patients [4]. The commonest causes are tension headache and migraine, which in combination are twenty times more common than PDPH, let alone the rarer complications of subdural hygromas and hematomas [5].

Subdural hygromas are composed of xanthochromic fluid and result from intracranial hypotension [6]. The prevailing theory is that cerebrospinal fluid (CSF) leaks into the epidural space via the dural defect leading to compensatory vasodilatation of the pachymeningeal blood vessels (Monro-Kellie doctrine), which subsequently become leaky [3, 7–10]. Some investigators have proposed that arachnoid granulation rupture may be a contributing factor [10]. Subdural hygromas occur in 10–69% of patients with intracranial hypotension and can occur as early as five hours or as late as five months after dural puncture [11–14].

If a dural tear is left untreated, continued spinal CSF leakage can lead to caudal sagging of the intracranial contents (occurring after ≥250 mL of CSF is lost) [15]. Traction-related tearing of subdural veins is the likely mechanism by which hygromas are complicated by hematomas, which may be unilateral or bilateral [14]. The risk of subdural hygroma and hematoma formation increases proportionally with the degree of intracranial hypotension and the number of dural punctures, as well as with coexistent cerebral atrophy, cerebral aneurysm, vascular malformation, pregnancy, dehydration, and use of anticoagulants.

The true incidence of subdural hematoma following dural puncture remains elusive as most patients are managed without imaging investigation. Studies have reported that, of the patients who develop subdural hygromas, 47% go on to develop subdural hematomas [16–18].

The cardinal feature of intracranial hypotension is an orthostatic headache, which is of variable quality, typically most severe within the first twenty-four hours and usually resolving within ten days [19, 20]. Altered conscious state, meningism, nausea, vomiting, dizziness, cranial nerve palsies, visual disturbance, photophobia, and rarely seizures have also been described [21]. Bradycardia has also been described and is thought to occur due to rostral migration of the brain with subsequent compression of the hypothalamus. Mass effect on the hypothalamus can cause alterations in autonomic outflow [22, 23].

If the headache persists, loses its postural nature, returns following initial resolution, or is associated with haemodynamic changes, neuroradiological investigation is advocated to assess sequelae of intracranial hypotension as a delay in diagnosis can be catastrophic [14]. Studies have demonstrated that dural puncture complicated by subdural hematoma carries a mortality rate of a value between 17 and 29% [14, 24].

Subdural fluid collections (hematomas or hygromas) can be managed safely with conservative methods, such as bed rest, hydration, and caffeine. If the patient is still symptomatic despite these measures, an epidural blood patch (EBP) should be performed [17]. Craniotomy or burr hole evacuation is rarely required even if the subdural fluid collection is large and exerts significant mass effect; however they may take up to three months to resolve [13, 25].

Anaesthetists need to be cognisant of the possibility of subdural hematomas in the setting of PDPH, especially in parturients experiencing persistent headache with neurological or haemodynamic disturbance. Early radiological investigation is encouraged, as a delay in diagnosis can be fatal.
